# Hypersensitive C-reactive protein as a potential indicator for predicting left ventricular hypertrophy in elderly community-dwelling patients with hypertension

**DOI:** 10.1186/s12872-023-03509-z

**Published:** 2023-09-27

**Authors:** Wei Song, Chunsheng Zhang, Jiamei Tang, Yan Li, Tiantian Jiao, Xueqi Lin, Yuanqi Wang, Jialiang Fang, Jingjing Sha, Tongjiu Ding, Jiayue Cheng, Jiming Li

**Affiliations:** 1 Department of Cardiology, Shanghai East Hospital, School of Medicine, Tongji University, Shanghai, 200092 China; 2 Department of General Practice, Jinyang Community Health Service Center, Shanghai, 200136 China; 3 Department of Ultrasound, Shanghai Changhai Hospital, Navy Medical University, Shanghai, China; 4 Department of Cardiology, Shanghai East Hospital, Tongji University School of Medicine, 150 Jimo Road, Pudong New Area, Shanghai, 200120 China

**Keywords:** Hypersensitive C-reactive protein, Left ventricular hypertrophy, Hypertension, Elderly patients

## Abstract

**Background:**

The aim of this study was to investigate the relationship between Hypersensitive C-reactive protein (hs-CRP) and left ventricular hypertrophy (LVH) in elderly community-dwelling patients with hypertension.

**Methods:**

A cross-sectional study was conducted, involving the recruitment of 365 elderly hypertensive residents ≥ 65 years of age from five communities. The participants were divided into two groups: an LVH group (n = 134) and a non-LVH group (n = 231), based on the left ventricular mass index (LVMI) determined by echocardiography. Spearman correlation analysis was used to assess the relationship between hs-CRP and LVH. Univariate and Multivariate analysis was performed to detect variables associated with LVH. The diagnostic value of hs-CRP for LVH was expressed as the area under the receiver operating characteristic (ROC) curve.

**Results:**

The incidence of LVH in elderly hypertension patients in the community was 36.7%. The hs-CRP levels were significantly higher in subjects with LVH compared to those without LVH (1.9 [0.8, 2.9] vs. 0.7 [0.4, 1.4], *P* = 0.002). Spearman correlation analysis demonstrated a positive correlation between hs-CRP and LVMI (*r* = 0.246, *P* < 0.001), as well as with IVST (*r* = 0.225, *P* < 0.001) and LVPWT (*r* = 0.172, *P* = 0.001). Among elderly hypertensive residents in the community, the cut-off value of hs-CRP for diagnosing LVH was 1.25 mg/L (sensitivity: 57.5%; specificity: 78.4%), and the area under the ROC curve for hs-CRP to predict LVH was 0.710 (95%*CI*: 0.654–0.766; *P* < 0.001). In the final model, hs-CRP ≥ 1.25 mg/L (OR = 3.569; 95%CI, 2.153–5.916; P<0.001) emerged as an independent risk factor for LVH. This association remained significant even after adjusting for various confounding factors (adjusted OR = 3.964; 95%CI, 2.323–6.765; P < 0.001).

**Conclusions:**

This community-based cohort of elderly hypertensive individuals demonstrates a strong association between hs-CRP levels and the presence of LVH. The hs-CRP ≥ 1.25 mg/L may serve as an independent predictor for LVH in hypertensive subjects and exhibit good diagnostic efficacy for LVH.

**Supplementary Information:**

The online version contains supplementary material available at 10.1186/s12872-023-03509-z.

Left ventricular hypertrophy (LVH) is a crucial manifestation of organ damage in hypertension [1]. Hypertension-induced LVH is an independent risk factor for heart failure, myocardial infarction, arrhythmias, sudden cardiac death, and stroke [2–4]. The prevalence of LVH in hypertensive patients have ranging from approximately 35–45% [5, 6]. Echocardiography is currently a commonly used tool for the diagnosis of LVH and is recommended by most guidelines [7, 8]. The development of LVH in hypertensive patients is believed to be primarily driven by haemodynamic changes and neural sympathetic activity caused by elevated blood pressure, leading to myocardial fibrosis, oxidative stress, and ischemia [2, 9, 10].

A growing body of evidence suggesting that LVH is a low-grade inflammatory disease. Several inflammatory markers, including hypersensitive C-reactive protein (hs-CRP), interleukin-6 (IL-6), tumor necrosis factor (TNF), neutrophil-to-lymphocyte ratio (NLR), and red blood cell distribution width (RDW), are closely associated with LVH in hypertensive patients [11–14]. Moreover, some of these markers have shown promising diagnostic efficacy for LVH. Of these, CRP has been extensively studied and is considered the most representative inflammatory factor. These findings have been demonstrated in studies conducted on specific hypertensive populations, such as those with newly diagnosed hypertension, pediatric hypertension, and hypertension combined with systemic diseases [15–17]. However, there is a lack of research focusing on the correlation between hs-CRP levels and LVH in elderly hypertensive patients residing in the community.

In this study, we conducted a cross-sectional investigation to examine the influence of hs-CRP on LVH in community-based elderly hypertensive individuals. Furthermore, considering its ease of measurement, hs-CRP has the potential to serve as a valuable biological marker for LVH screening in the community.

## Methods

### Study population

We selected elderly hypertensive patients aged ≥ 65 years registered in the resident health record from five communities (Jinyang No.6 Village, Luoshan No.6 Village, Xiangshan No.5 Village, Jintai No.2 Village, and Qinningsi Village) of Jinyang Subdistrict in Pudong New Area of Shanghai. All subjects were enrolled in annual health screening from June to December 2021. Inclusion included both currently treated and untreated patients with hypertension. The long-term reference for the diagnosis of hypertension in the community is the criterion of a systolic blood pressure ≥ 140 mmHg or a diastolic blood pressure ≥ 90 mmHg. The exclusion criteria were as follows: (1) presence of acute or chronic infectious disease, rheumatoid immune system disease, acute cardiovascular disease, malignancy, severe heart failure, severe liver and kidney insufficiency, taking steroid hormone drugs, radiological injury, and various surgeries and traumas that may lead to increased hs-CRP; (2) presence of atrial fibrillation, heart valve disease or hypertrophic cardiomyopathy that may cause cardiac hypertrophy; (3) previously diagnosed diseases that can be secondary to hypertension such as hyperthyroidism, aortic stenosis, aldosteronism, renal artery stenosis, pheochromocytoma;(4) presence of severe intellectual impairment, cognitive impairment, mental impairment or motor dysfunction that may affect the assessment. All enrolled patients signed informed consent forms. The Ethics Committee of Jinyang Community Health Service Center approved the study.

### Data Collection and Analysis

Data were collected using a structured questionnaire. These items included age, gender, smoking history, previous medical history, and current medication. Height, weight, and hip circumference were measured to obtain body mass index (BMI) and waist-to-hip ratio (WHR). Transient blood pressure was measured twice in an inactive state at an interval of 5 min, and the average of the two measurements was taken. If the difference in systolic or diastolic blood pressure was more than 5mmHg between the two times, it should be measured again, and the average of the three times should be taken. They were fasting in the early morning on the day of the health screening. Blood count, blood glucose, total cholesterol, triglycerides, low-density lipoprotein (LDL) cholesterol, and creatinine will be measured on the same day. If the subject meets the requirements of this study, additional hs-CRP testing will be required. The testing machine is Roche Cobas c702 fully automatic biochemical analyzer, and the reagent is Roche reagent. The method is immunoturbidimetry. Professional laboratorians performed all the tests.

### Echocardiography and definition of LVH

An experienced ultrasound specialist performed the echocardiography. Cardiac structures were measured by Philip Affiniti30 color Doppler ultrasound with a probe frequency of 2–4 MHZ. The subjects were placed in the left lateral decubitus position. The probe was placed between the left sternal border of 3 and 4 ribs, perpendicular to the chest wall, in the parasternal left ventricular long axis section, at the level of the mitral chordate tendineae. The main measurements included left ventricular end-diastolic diameter (LVEDD), interventricular septal thickness (IVST), and left ventricular posterior wall thickness (LVPWT) on an M-mode image. Two qualified physicians verified all measurements. Three consecutive cardiac cycles were measured, and the mean value was obtained. Left ventricular mass (LVM) was normalized to left ventricular mass index (LVMI) by body surface area (BSA) according to the new American Society of Echocardiography (ASE) criteria [18]. LVM (g) = 0.8 × 1.04 × [(LVEDD + IVST + LVPWT)^3^-LVEDD^3^] + 0.6. LVMI = LVM (g) /ABS (m^2^). LVH is defined as LVMI ≥ 115 g/m^2^ in men and LVMI ≥ 95 g/m^2^ in women [8].

### Statistical analyses

Continuous variables with a normal distribution were described as mean ± standard deviation (SD) and non-normally distributed as the median and interquartile range (IQR). Categorical variables were presented as the number of observations and percentage. Differences between the two groups were calculated using the *t* test, Wilcoxon rank sum, or χ2 test as appropriate. Spearman correlation was used to test correlations between hs-CRP level and echocardiographic data. The diagnostic value of hs-CRP for LVH was expressed as the area under the receiver operating characteristic (ROC) curve. Univariate analysis was performed to detect variables associated with LVH. Three inflammatory markers (hs-CRP, NLR, RDW) were used for multiple logistic regression with variables with P < 0.1 in univariate regression, respectively. Statistical analysis was performed using SPSS statistical software (SPSS version 26.0, IBM, Armonk, USA). The level of significance was set at *P* < 0.05.

## Results

Of the 487 selected elderly hypertensive subjects who attended the annual health screening program, 365 subjects consented to participate in the study and underwent hs-CRP testing and echocardiography. The mean age was 71.88 ± 4.96 years, with 54.0% being female. Among them, 134 subjects (36.7%) were diagnosed with LVH. The baseline characteristics of the LVH and non-LVH groups are shown in Table 1. Individuals with LVH had a longer duration of hypertension and a higher mean SBP than subjects without LVH. The proportion of women was higher in the LVH group, whereas smoking was correspondingly lower. Additionally, individuals with LVH had a higher mean body mass index (BMI). No significant differences in other risk factors and current medications between the two groups. In addition to LVMI, other commonly used quantitative indicators of LVH, such as IVST and LVPWT, were significantly higher in the LVH group than in the non-LVH group.

**Table 1:** Comparison of baseline characteristics between hypertension with LVH group and Non-LVH group in elderly community participants

Characteristics	LVH (n = 134)	Non-LVH (n = 231)	*P* value
Age (y), mean ± SD	73.00 ± 5.47	71.23 ± 4.52	0.001
Female, n (%)	95 (70.9)	102 (44.2)	<0.001
Duration of hypertension (y), median (IQR)	10 (5.75, 20)	8 (4, 15)	0.001
SBP (mmHg), mean ± SD	144.04 ± 19.30	139.93 ± 17.87	0.040
DBP (mmHg), mean ± SD	81.54 ± 9.58	81.64 ± 10.25	0.927
MABP (mmHg), mean ± SD	102.37 ± 11.13	100.95 ± 11.50	0.249
Diabetes mellitus, n (%)	29 (21.6)	35 (15.1)	0.108
Dyslipidemia, n (%)	47 (35.1)	68 (29.4)	0.264
Coronary artery disease, n (%)	16 (11.9)	25 (10.8)	0.744
Previous stroke, n (%)	14 (10.4)	12 (5.2)	0.060
History of smoking, n (%)	40 (28.9)	93 (40.3)	0.046
BMI (kg/m2), mean ± SD	24.83 ± 3.71	23.62 ± 2.78	<0.001
WHR, mean ± SD	0.87 ± 0.05	0.87 ± 0.07	0.587
Current medications, n (%)			
ACE inhibitor	8 (6.0)	22 (9.5)	0.233
ARB	65 (48.5)	93 (40.3)	0.125
Beta blocker	17 (12.9)	31 (13.4)	0.842
Calcium channel blocker	85 (63.4)	134 (58.0)	0.308
Hydrochlorthiazide	25 (18.7)	35 (26.5)	0.384
Statin or other lipid lowering agent	41 (30.6)	55 (23.8)	0.156
Aspirin or other antiplatelet agent	30 (22.4)	42 (18.2)	0.330
Insulin or oral hypoglycaemic agents	26 (19.4)	34 (14.7)	0.232
Total cholesterol (mmol/dL), mean ± SD	5.11 ± 1.04	5.13 ± 0.99	0.888
LDL- cholesterol (mmol/dL), mean ± SD	2.98 ± 0.82	2.99 ± 0.84	0.924
Triglyceride (mmol/dL), mean ± SD	1.73 ± 0.91	1.66 ± 0.87	0.417
Fasting glucose (mmol/L), median (IQR)	6.0 (5.4, 6.9)	5.7 (5.3, 6.5)	0.031
Serum creatine (umol/L), median (IQR)	75 (65, 87)	80 (71, 91)	0.026
hs-CRP (mg/L), median (IQR)	1.9 (0.8, 2.9)	0.7 (0.4, 1.4)	0.002
NLR, median (IQR)	1.87 (1.40, 2.33)	1.73 (1.34, 2.67)	0.218
RDW (%), median (IQR)	13.34 ± 0.79	13.30 ± 0.91	0.691
LVMI (g/m2), median (IQR)	116.82 (101.74, 129.28)	86.35 (77.70, 93.53)	<0.001
IVST (mm), median (IQR)	12 (11, 12.25)	10 (9, 11)	<0.001
LVPWT (mm), mean ± SD	11 (10,11)	9 (9, 10)	<0.001

SBP: systolic blood pressure; DBP: diastolic blood pressure; MABP: mean arterial blood pressure; BMI: body mass index; WHR: waist-to-hip ratio; LDL: low-density lipoprotein; ACE: angiotensin converting enzyme; ARB: angiotensin receptor blockers; hs-CRP: hypersensitive C-reactive protein; NLR: neutrophil-to-lymphocyte ratio; RDW: red blood cell distribution width; LVMI: left ventricular mass index; IVST: interventricular septal thickness; LVPWT: left ventricular posterior wall thickness

Subjects with LVH exhibited higher hs-CRP levels compared to those without LVH (1.9 [0.8, 2.9] vs. 0.7 [0.4, 1.4], *P* = 0.002). The hs-CRP level of the LVH and non-LVH groups is presented in Table 1. The hs-CRP level was positively correlated with LVMI (*r* = 0.246, *P*<0.001), IVST (*r* = 0.225, *P*<0.001), and LVPWT (*r* = 0.172, *P* = 0.001). NRL and RDW did not significantly differ between LVH and non-LVH groups. However, the RDW level exhibited a positive correlation with LVMI (*r* = 0.135, *P* = 0.010), IVST (*r* = 0.848, *P* = 0.001), and LVPWT (*r* = 0.194, *P*<0.001). There was no significant correlation between NRL and echocardiographic parameters.The data are presented in Table 2.

**Table 2:** Correlation of inflammatory markers and parameters

	hs-CRP		NLR		RDW	
	*r*	*P*	*r*	*P*	*r*	*P*
Age	0.145	0.005	0.073	0.163	0.100	0.057
Duration	0.052	0.318	0.020	0.705	0.155	0.003
SBP	0.059	0.262	0.068	0.192	0.049	0.349
BMI	0.284	<0.001	0.025	0.629	0.073	0.163
Fasting glucose	0.088	0.094	0.140	0.007	0.015	0.774
Serum creatine	0.061	0.248	0.050	0.338	0.103	0.049
LVMI	0.246	<0.001	0.069	0.187	0.135	0.010
IVST	0.225	<0.001	0.095	0.069	0.848	0.001
LVPWT	0.172	0.001	0.077	0.141	0.194	<0.001

SBP: systolic blood pressure; BMI: body mass index; LVMI: left ventricular mass index; IVST: interventricular septal thickness; LVPWT: left ventricular posterior wall thickness

The hs-CRP cut-off value for diagnosing LVH in the ROC curve was determined to be 1.250 mg/L (sensitivity: 57.5%; specificity: 78.4%), with an AUC was 0.710 (95% *CI*: 0.654–0.766; *P* < 0.001). The sensitivity of NLR and RDW in diagnosing LVH was 90.3% and 66.4%, respectively, while the specificity was 19.5% and 43.7%, respectively. The three inflammatory indicators were dichotomized based on their cut-off values, and separate multiple regression analyses were conducted in Supplementary Tables 1–3. The results of the multivariate logistic regression are presented in Table 3. In the final model, hs-CRP ≥ 1.25 mg/L (*OR* = 3.569; 95%*CI*, 2.153–5.916; *P*<0.001) emerged as an independent risk factor for LVH. This association remained significant even after adjusting for various confounding factors (adjusted *OR* = 3.964; 95%*CI*, 2.323–6.765; *P* < 0.001). Additionally, after adjustment, an NLR ≥ 1.21 also demonstrated predictive value for LVH (*OR* = 2.187; 95%*CI*, 1.038–4.608; *P* = 0.040), while RDW did not.

**Table 3:** Multivariable analysis of the three inflammatory factors for LVH separately

Characteristics	Multivariable analysis				
	Unadjusted^*^OR (95%*CI*)	*P* Value		Adjusted^**^OR (95%*CI*)	*P* Value
hs-CRP ≥ 1.25 mg/L	3.569 (2.153, 5.916)	<0.001		3.964 (2.323, 6.765)	<0.001
NLR ≥ 1.21	2.397 (1.157, 4.964)	0.019		2.187 (1.038, 4.608)	0.040
RDW ≥ 13.05%	1.561 (0.941, 2.591)	0.085		1.448 (0.863, 2.429)	0.161

hs-CRP: hypersensitive C-reactive protein; NLR: neutrophil-to-lymphocyte ratio; RDW: Red blood cell distribution width;

* Multivariate logistic regression was conducted for variables that reached *P* < 0.1 following univariate analysis: age, gender, duration of hypertension, SBP, previous stroke, smoking, BMI, fasting glucose, serum creatine

** The adjustment variables included age, gender, duration of hypertension, SBP, diabetes mellitus, dyslipidemia, coronary artery disease, previous stroke, smoking, BMI, WHR, current medications, fasting glucose, serum creatine

## Discussion

This community-based cohort involving elderly hypertensive subjects reveals a strong association between hs-CRP and the presence of LVH. The level of hs-CRP is positively correlated with the severity of LVH. Furthermore, hs-CRP serves as an independent predictor of LVH in hypertensive subjects and exhibits a good diagnostic efficacy for LVH.

A growing body of evidence suggests that low-grade inflammation plays a significant role in the development of hypertension and its associated complications [12, 19, 20]. In particular, LVH is strongly associated with inflammation. Previous studies have demonstrated that CRP, NLR, and RDW can increase the risk of LVH in hypertensive patients [11, 13–15, 21, 22]. In our study, we compared the routine indicators of inflammation between the two groups using the health screening program. However, except for hs-CRP, RDW and NLR were not significantly elevated in the LVH group, which is inconsistent with previous findings [14, 21, 22]. While hs-CRP and NLR were independently correlated with LVH, RDW did not show an independent influence on LVH in our study. These results differ from previous studies [15, 22]. One possible explanation is that the previous studies included newly diagnosed or untreated hypertension patients, where the inflammatory effects were not suppressed. In our cohort, a large proportion of this cohort had a long duration of hypertension and currently had relatively low blood pressure, which might contribute to the stable CRP secretion. CRP has been widely used as a marker of systemic inflammation, and measuring hs-CRP in serum is a sensitive method to improve the accuracy of CRP detection. As an important inflammatory factor, hs-CRP has consistently shown a significant association with LVH in past and present studies. Salles et al. [12] reported that high CRP (≥ 3.7 mg/L) is independently associated with the occurrence of LVH in patients with resistant hypertension, even after adjusting for important confounding factors. Studies conducted by Seyfeli et al. [15] and Yu et al. [11] consistently demonstrated a significant association between serum hs-CRP and left ventricular diastolic function as well as concentric hypertrophy in hypertensive patients. The precise pathophysiological mechanisms underlying these associations are still not fully understood. Studies in CRP transgenic mice have shown that CRP exacerbates pressure overload-induced cardiac remodeling through enhanced inflammatory response and oxidative stress [23]. Furthermore, cardiac remodeling in these mice was significantly aggravated after angiotensin II (Ang II) infusion, resulting in reduced left ventricular ejection fraction and increased cardiac fibrosis [24]. This phenomenon is thought to be associated with CRP-mediated upregulation of Ang II type I receptor expression and activation of the transforming growth factor-β/Smad and nuclear factor-κB signaling pathways [24].

Studies have consistently demonstrated that age, gender, obesity, and other factors also have varying degrees of influence on LVH, which has been widely established in previous research [25–27]. CRP, being a non-specific marker, can be elevated secondary to infection, trauma, and inflammation. Additionally, it is influenced by age and gender, obesity, chronic diseases. Apart from increasing the risk of LVH, these factors can also elevate CRP levels. In this study, it was also found that hs-CRP levels were positively correlated with age and BMI. Previously, several studies have confirmed a significant increase in CRP levels among individuals with high BMI [28, 29], attributed to the production of interleukin-6 by visceral adipose tissue, which stimulates CRP secretion by the liver [30, 31]. In our cohort, subjects with LVH exhibited a higher proportion of risk factors such as obesity and previous stroke. It is plausible that these risk factors, in conjunction with elevated CRP levels, may have synergistic effects in the development of LVH, potentially serving as underlying pathophysiological mechanisms. The prevention of traditional risk factors, particularly obesity, may be instrumental in mitigating the low-grade inflammatory mechanisms underlying hypertension.

In addition, a growing body of research suggests that various types of antihypertensive, glucose-lowering and statins have positive effects on LVH to varying degrees [32–35]. They can exert cardioprotective effects by reversing LV remodelling and altering LV structure. Of course, these drugs themselves also have some inhibitory effect on the inflammatory response, thus reducing CRP secretion [35, 36]. In this cohort, there was no difference in the proportions of various medicines between the two groups, and even a slightly higher proportion of some drugs were used in the LVH group, such as statins and ARB antihypertensive drugs. This is mainly due to the fact that the LVH group has a more difficult to control blood pressure and a higher proportion of comorbidities, which makes it difficult to show the protective effect of drugs. However, the drug factor cannot be ignored, and it was fully taken into account in the regression analyses.

The standard diagnostic methods of LVH include electrocardiogram, echocardiography, and cardiac magnetic resonance (CMR). These methods vary in terms of sensitivity, specificity, and accessibility. Among them, the electrocardiogram is recommended as the screening method for LVH in the community hypertensive population due to its simplicity and repeatability [8]. It has high diagnostic specificity, particularly for patients with severe LVH, where specificity can reach 80-90%. However, it has limitations in terms of diagnostic sensitivity, with values of 7–35% for mild LVH and 30–60% for moderate to severe LVH [37]. In this study, we evaluated the diagnostic efficacy of different simple inflammatory indicators and found that only hs-CRP exhibited for LVH. This finding contrasts with the study by Yu et al. [11], where NLR was found to be independently associated with LVH in hypertensive patients, consistent with the diagnostic efficacy of CRP. However, in our study, NLR did not demonstrate significant diagnostic value for LVH. Only hs-CRP consistently showed an advantage, with an ROC of 0.702 for LVH diagnosis. Although the diagnostic sensitivity of hs-CRP in both studies was below 60%, it still represents a significant improvement over the electrocardiogram. Thus, hs-CRP may serve as a potential inflammatory marker for screening LVH in the hypertensive population in community settings.

Some limitations of this study should be acknowledged and considered. Firstly, the sample size was relatively small, which may introduce bias and limit the generalizability of the findings. Additionally, there are several factors that can influence LVH, and the study may not have fully adjusted for all potential confounding variables. Regarding the hs-CRP index itself, its stability across different detection methods and environments may not be ideal, highlighting the need for establishing unified norms and standards. Furthermore, more validation studies are warranted in the future to further confirm the findings of this study.

In conclusion, hs-CRP levels were found to be significantly increased in the elderly community hypertensive population with LVH. The hs-CRP, being a simple, relatively inexpensive, and universally available test. Increased hs-CRP levels can be used as a reference for echocardiography in community screening for LVH. Its use could facilitate the monitoring of cardiac remodeling and enable early intervention to reduce the incidence of heart failure.

### Electronic supplementary material

Below is the link to the electronic supplementary material.

Supplementary Material 1

## Data Availability

The datasets generated for this study are available on request to the corresponding author.

## Abbreviations

hs-CRP: Hypersensitive C-reactive protein (hs-CRP)
LVH: left ventricular hypertrophy
SBP: systolic blood pressure
DBP: diastolic blood pressure
MABP: mean arterial blood pressure
BMI: body mass index
WHR: waist-to-hip ratio
LDL: low-density lipoprotein
NLR: neutrophil-to-lymphocyte ratio
RDW: red blood cell distribution width
LVMI: left ventricular mass index
IVST: interventricular septal thickness
LVPWT: left ventricular posterior wall thickness
