# Supplementary material for: Hypersensitive C-reactive protein as a potential indicator for predicting left ventricular hypertrophy in elderly community-dwelling patients with hypertension
Source: BMC Cardiovasc Disord. 2023 Sep 27;23:480. doi: 10.1186/s12872-023-03509-z (PMC10537845; doi:10.1186/s12872-023-03509-z)
Supplement: Supplementary file 1 — Supplementary Material 1 [file 12872_2023_3509_MOESM1_ESM.docx]

**Table S1. Multiple logistic regression of hs-CRP and variables with *P* < 0.1 in univariate regression for LVH in elderly hypertensive patients in the community**

| Characteristics | OR (95% CI) | *P* value |
| --- | --- | --- |
| Age | 1.088 (1.032, 1.148) | 0.002 |
| Female | 5.139 (2.509, 10.523) | ＜0.001 |
| Duration of hypertension | 1.042 (1.012, 1.073) | 0.005 |
| SBP | 1.003 (0.989, 1.017) | 0.678 |
| Previous stroke | 1.322 (0.506, 3.450) | 0.569 |
| History of smoking | 1.578 (0.838, 2.972) | 0.158 |
| BMI | 1.074 (0.991, 1.165) | 0.081 |
| Fasting glucose | 1.143 (0.998, 1.309) | 0.054 |
| Serum creatine | 0.996 (0.979, 1.014) | 0.693 |
| hs-CRP≥1.25mg/L | 3.569 (2.153, 5.916) | ＜0.001 |

SBP: systolic blood pressure; BMI: body mass index; hs-CRP: hypersensitive C-reactive protein.

# Table S2 **Multiple logistic regression of NLR and variables with *P* < 0.1 in univariate regression for LVH in elderly hypertensive patients in the community**

| Characteristics | OR (95% CI) | *P* value |
| --- | --- | --- |
| Age | 1.097 (1.041, 1.156) | ＜0.001 |
| Female | 5.677 (2.827, 11.398) | ＜0.001 |
| Duration of hypertension | 1.037 (1.009, 1.066) | 0.009 |
| SBP | 0.999 (0.985, 1.012) | 0.832 |
| Previous stroke | 1.438 (0.573, 3.611) | 0.439 |
| History of smoking | 1.617 (0.878, 2.980) | 0.123 |
| BMI | 1.122 (1.036, 1.216) | 0.005 |
| Fasting glucose | 1.128 (0.990, 1.285) | 0.070 |
| Serum creatine | 0.998 (0.980, 1.016) | 0.803 |
| NLR≥1.21 | 2.397 (1.157, 4.964) | 0.019 |

SBP: systolic blood pressure; BMI: body mass index; NLR: neutrophil-to-lymphocyte ratio.

# Table S3 **Multiple logistic regression of RDW and variables with *P* < 0.1 in univariate regression for LVH in elderly hypertensive patients in the community**

| Characteristics | OR (95% CI) | *P* value |
| --- | --- | --- |
| Age | 1.091 (1.036, 1.149) | 0.001 |
| Female | 5.318 (2.667, 10.601) | ＜0.001 |
| Duration of hypertension | 1.033 (1.005, 1.062) | 0.023 |
| SBP | 1.000 (0.987, 1.014) | 0.953 |
| Previous stroke | 1.311 (0.521, 3.303) | 0.565 |
| History of smoking | 1.511 (0.817, 2.793) | 0.188 |
| BMI | 1.129 (1.041, 1.224) | 0.004 |
| Fasting glucose | 1.155 (1.011, 1.319) | 0.034 |
| Serum creatine | 0.995 (0.977, 1.012) | 0.544 |
| RDW≥13.05% | 1.561 (0.941, 2.591) | 0.085 |

SBP: systolic blood pressure; BMI: body mass index; RDW: Red blood cell distribution width.
